# Characterisation of cuticle mechanical properties: analysing stiffness in layered living systems to understand surface buckling patterns

**DOI:** 10.1039/d4sm01406e

**Published:** 2025-10-08

**Authors:** Chiara A. Airoldi, Chao Chen, Humberto Herrera-Ubaldo, Hongbo Fu, Carlos A. Lugo, Udhaya Ponraj, Alfred J. Crosby, Beverley J. Glover

**Affiliations:** a Department of Plant Sciences, University of Cambridge Downing Street Cambridge CB2 3EA UK bjg26@cam.ac.uk; b Polymer Science and Engineering Department, University of Massachusetts Amherst Amherst MA 01003 USA acrosby@umass.edu; c Sainsbury Laboratory, University of Cambridge Cambridge CB2 1LR UK; d Department of Biosciences, Sri Sathya Sai Institute of Higher Learning Anantapur Andhra Pradesh - 515001 India

## Abstract

Development of a living organism is a highly regulated process during which biological materials undergo constant change. *De novo* material synthesis and genetically-regulated changes in mechanical properties of materials are key for organ development. However, few studies have attempted to produce quantitative measurements of the mechanical properties of biological materials during growth. Such quantitative analysis is particularly challenging where the material is layered, and yet layering of materials with different mechanical properties may be essential to morphogenetic pattern formation. This is the case for the *Hibiscus trionum* flower petal, where buckling of the cuticle on top of the epidermal cell wall forms ridges, producing an iridescent effect. This ridge formation is hypothesised to be due to mechanical instability, which directly depends upon the mechanical properties of the individual layers of cuticle and cell wall. We set out to develop methods to measure the mechanical properties of the surface layers of plant epidermal cells through atomic force microscopy (AFM). To ensure that our results were reproducible and represented the most appropriate combination of experimental parameters, we used the uniaxial tensile tester for ultrathin films (TUTTUT) to provide independent measurement of cuticle stiffness. We explored mechanical properties of the upper cuticle and lower cuticular layer of the epidermal cell surface across growth stages. In addition to offering technical approaches to explore the stiffness of living layered materials, our findings suggest that temporal changes in biological material properties are key to understanding the development of biological surface patterns.

## Abstract 2

Development of a living organism is a highly regulated process during which biological materials undergo constant change.

## Graphical abstract

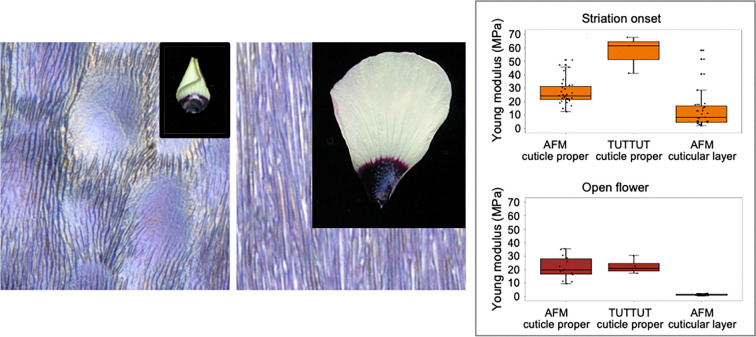

## Introduction

*Hibiscus trionum* petals display beautiful colours, which are produced by pigments and structural features. In the purple pigmented regions, the cuticle presents elongated rows of nano-ridges on top of semi-flat cells, which form a semi-ordered diffraction grating.^1^ The diffraction of the light by these ridges creates a weak iridescence with a scattered blue halo effect that is perceived by bees and can increase the salience of the flowers to foraging pollinators.^2^ The petal surface forms a bilayer structure, with the extracellular cuticle proper (composed primarily of cutin and waxes) sitting on top of a cell wall-based cuticular layer (composed of cellulose impregnated with cutin monomers). It has been shown using dissected petal samples combined with continuum mechanical theories that mechanical buckling is sufficient to explain the formation of the *Hibiscus trionum* petal cuticular striations.^3,4^

We have recently explored the theoretical basis of mechanical buckling in the *Hibiscus trionum* system.^4^ According to linear elastic buckling theory, a bilayer system with a stiff thin film resting on a soft substrate will form a periodic wrinkle pattern when an in-plane compressive strain exceeds a critical threshold value, *ε*_c_. If the substrate thickness is much larger than the film thickness, the wavelength, *λ*, at the onset of a wrinkled pattern in an incompressible material is determined by:^5^1
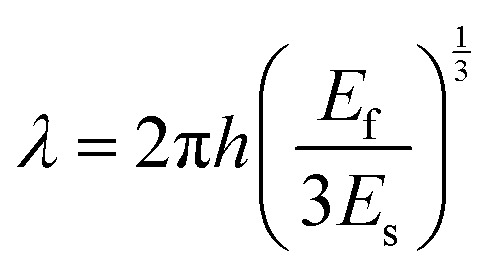
where *E*_f_ and *E*_s_ are the Young's moduli of the film and the substrate, and *h* is the film thickness. While this instability process and predicted wavelength is qualitatively consistent with the structural development of the *Hibiscus trionum* petal system, detailed quantitative connections have yet to be established due to the difficulty of measuring the mechanical properties of the structures involved. Furthermore, it has been shown that the application of similar forces alone cannot cause buckling of similar petal samples that do not normally form cuticular striations.^3^ These observations suggest that the mechanical properties of the *Hibiscus trionum* system, as well as possible changes during growth, may play important roles in the formation of these surface nanostructures that have ecological importance.

Our next step is to analyse the Young's moduli of the film and substrate and the film thickness as the petal structures grow. These data would allow us to determine whether regions of the *H. trionum* petal that do produce striations have mechanical properties that fit the model and, conversely, whether regions of the petal that do not buckle have mechanical properties that preclude buckling. In addition, the ability to measure these parameters would allow us to test the model by perturbing petal mechanical properties and exploring the consequences for pattern formation. Such experiments might involve pharmacological or genetic manipulation of tissue properties and behaviour. However, analytical techniques that provide direct insight into the mechanical properties of layered living systems with minimal intervention are currently unavailable.

In biological systems, the technique of choice for measuring surface stiffness is atomic force microscopy (AFM). AFM has been adapted for use with a number of biological materials, more commonly with animal systems than with plants.^6,7^ There are numerous challenges, including the inherent instability of dissected living tissue. With plant material this challenge is exacerbated by the highly vacuolate nature of epidermal cells, dependent on turgor for cell integrity and morphology. However, of most relevance for our current study is that all such previous studies treat the plant surface as a single material, rather than a series of layers with the potential to exhibit different mechanical properties. For example, analyses of surface stiffness of algal thallus, plant leaves, apical meristems or plant cells in culture all simply report a single Young's modulus.^8–11^ Since all aerial plant organs are covered by an extracellular cuticle, these approaches are at a minimum underestimating the complexity of the material described. In our case, studying a system in which the bilayered nature of the material is hypothesised to be intrinsic to pattern formation, they fail to address the key question of the relative stiffness of the distinct layers.

In addition to a need to understand living bilayers, knowledge of how the mechanical properties of materials change during growth would aid our understanding of how specific wrinkle patterns and dimensions are achieved during petal development. While it seems intuitively likely that the mechanical properties of biological materials change during development, as gene expression profiles change and tissue chemistry is altered, very few studies have been dedicated to measuring such changes in living, growing tissues.

In this work, we developed tools to measure the mechanical properties of the cuticle proper (the surface layer) and the cuticular layer (the substrate layer) on the surface of petals from the onset of surface buckling to the fully developed flower. We used two independent methods, atomic force microscopy (AFM) and the uniaxial tensile tester for ultrathin films (TUTTUT). AFM is accessible to many biology labs, while TUTTUT is harder to access, but the use of the two techniques, in separate labs with separate experimentalists (and on different continents), allowed us to explore the reproducibility of our AFM data and provide confidence in our findings. By directly characterising the material and geometric properties of *H. trionum* petals, we were able to assess whether the morphology we observe can be predicted by mechanical theory. We found that the properties of the tissue at the start of buckling can predict the wrinkling initiation strain and the wrinkle wavelength. We also found that the pattern wavelength is set immediately following initiation, such that the later development of the petal does not substantially change the pattern wavelength, perhaps due to the inelasticity of biological materials during growth. The introduction of TUTTUT further provides pathways for exploring the cuticle mechanical response at larger strains, beyond where AFM characterisation is appropriate. The approach described here for measurement of mechanical properties of a living bilayer during development will provide new insights in the field of biomechanics and facilitate greater understanding of biological pattern formation.

## Materials and methods

### Plant growth conditions

*Hibiscus trionum* seeds were obtained from Cambridge University Botanic Garden. Plants were grown in Levington's M3 compost under a 16 hour daylight regime, in Cambridge in a greenhouse with a controlled temperature of 21 °C and in Amherst in a greenhouse with a controlled temperature of 23 °C. Buds were measured using a Vernier caliper after removing the epicalyx and measuring from the base of the bud to the top.

### Bright field light microscopy

A VHX-5000 Keyence microscope with a VH-Z500R/W/T 392 objective was used for bright field microscopy.

### Atomic force microscopy (AFM)

An AFM JPK NanoWizard with a cantilever tip of tetrahedral shape (ATEC-CONT Au10 S/N: 74454F6L1180 Nanosensor; nominal values: length 450 μm, mean width 50 μm, thickness 2 μm; Fig. S1) was used. This tip was chosen because of its small half cone angle that increases its performance on samples with a small pattern size combined with steep sample features. Three cantilevers were used over the course of the experiments whose results are described here. The cantilever manufacturer provided that the range of spring constants was 0.02–0.75 N m^−1^. To provide as much accuracy as possible, we calibrated each cantilever's spring constant before use with the thermal noise method in air.^12^ We measured the individual spring constants as 0.25 N m^−1^, 0.16 N m^−1^ and 0.26 N m^−1^, each within the manufacturer's provided range. Sensitivity was calculated in water at the beginning of every set of measurements by fitting the slope of a force-displacement curve acquired on a stiff substrate (a glass slide).^13^ The measured values ranged from 50 nm V^−1^ to 80 nm V^−1^.

Samples were prepared by positioning a small square of the peeled epidermis on a glass slide. We tested the effect of surrounding the sample with 1% low melting point agarose and of positioning it on a cooled but not completely solidified 3% agarose gel. No differences were observed in our measurements between the two methods, but the 3% agarose reduced movement of the samples and was thus used for all measurements reported here. Measurements were conducted in water with sucrose (0.005 M), dropped directly onto the sample.^13^

We measured the Young's modulus of the cuticle proper (upper layer) on top of intact petal surface. We measured the cuticular layer (substrate) following removal of the cuticle proper (upper layer) using a sharp and flexible blade while monitoring the process under a stereomicroscope.

We performed two types of measurements. We used the JPK software (JPK00806) in force mapping mode. Force-displacement curves were recorded within an area of 1 μm × 1 μm with a grid of 32 × 32 points (Fig. S2). Force–displacement curves were recorded at a speed of 40 μm s^−1^. The JPK Imaging mode was used to acquire images of the petals. The area scanned was 20 μm × 20 μm with a grid of 32 × 32 pixels. The tip velocity was 13.4 μm s^−1^, and a set point of 5 nN was used. The depth of the indentations was never more than 1/3 of the depth of the layer measured, based on cryo scanning electron microscopy imaging from previous analyses.^4^ These analyses showed that the thickness of the cuticle proper is 146 nm at stage 3 and 193 nm at stage 5, while the thickness of the cuticular layer is 2544 nm at stage 3 and 1879 nm at stage 5 (Table 1). For all the analyses, all the stages, cuticle proper and cuticle layers, we used a cut-off value of 10 nm.

**Table 1:** Summary of cellular features and cuticle material properties measured using AFM used to calculate the wavelength values using eqn (1). Values reported in Lugo *et al.* 2023 are marked with*

Sample	Thickness			*E*, Young modulus			Wavelength	
				(TUTTUT)	(AFM)			
	Cuticle proper*	Cut. layer top*	Cut. layer valley*	Cuticle proper	Cuticle proper	Cuticular layer	Theoretical	Observed*
	nm	nm	nm	MPa	MPa	MPa	nm	nm
Pre-striation	89.0	1790.8	1790.8					
Onset	146.3	2544.2	2429.9	56.90	27.06	13.65	799.0 (±363.6)	1308.8
Mature bud	210.0	2605.0	2171.6		21.64	4.03	1602.0	1289.3
Open flower	193.0	1879.9	1473.4	22.40	21.57	1.35	2117.1	1313.8

Images were exported directly from the cantilever height measured without a plane or line subtraction to give a better idea of the overall shape of the petal surface. Measurements were made on at least three different regions within a petal, and three measurements per region were performed: total sample numbers are indicated in Fig. 3.

### AFM data analysis

JPK SPM Data Processing BRUKER software 6.4.-1+ (JPK Bruker) was used for data analysis.^13,14^*F*–*d* curves were processed with the following operations (Fig. S3 and S4): subtraction of the baseline offset (baseline subtraction; offset + tilt subtraction using the best fit line over the range from *X*_min_ = 90%, *X*_max_ = 100% of the curve); adjustment of the *X*_offset_ (determination of contact point, the first crossing of zero force line); correction of the height of the bending of the cantilever; and the cantilever deflection is subtracted from the piezo height (vertical tip position correction). The trendline fit to determine the elastic modulus was calculated on the extend, or loading, portion of the curves. Zemla *et al.*^15^ showed that pyramidal shapes fit for a pyramidal tip do not give accurate Young's moduli values. The choice of contact mechanics model was determined considering the linear scaling of the measured *F*–*d* relationships in the first 10 nm of indentation, as well as microscopy analysis of the SPM tips. A flat cylinder contact mechanics model^16^ with a fixed contact radius (radius = 10 nm, see Fig. S1 for tip details) was applied to extract Young's moduli values (JPK Data Processing User Manual, Version 8.1 – 04/2024, Bruker Nano GmbH), assuming a Poisson ratio of 0.5 and applying the fitting range from −10 nm to 0 nm (X absolute position).
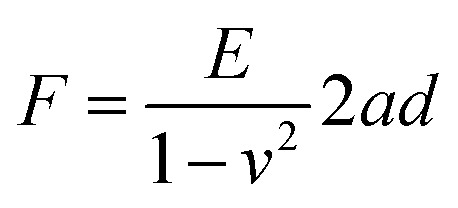
*F* = force, *E* = Young's modulus, *ν* = Poisson's ratio, *d* = indentation (vertical tip position), *a* = radius of contact

### TUTTUT

To assess whether our AFM approach gave reliable values for stiffness of the upper layer (the cuticle proper), despite it being still subtended by the cuticular layer, we used an alternative approach to explore the Young's modulus of isolated cuticle proper. TUTTUT requires specimens of several millimetres in width and length, so the peeled cuticle removed using a sharp blade could not be used, as the specimen fragments on removal. Therefore, the cuticle proper was separated from *H. trionum* petals using an enzymatic protocol modified from a previous method of cuticle isolation from tomatoes.^17,18^ In brief, petals were treated in an aqueous enzyme bath consisting of fungal cellulase (0.2% w/v), pectinase (2.0% w/v), and sodium azide (1 mM) in sodium citrate buffer (pH 3.7, Millipore Sigma Corporate). Petals were placed on a glass plate, and the abaxial side of the petals was scratched off with a razor blade such that the components beneath the adaxial cuticle were exposed for enzymatic treatment. The petal was submerged in the enzyme solution in an enclosed container to avoid evaporation and incubated at 35 °C for 24 hours. The isolated cuticle was then moved using tweezers to a glass slide and rinsed with deionized water. We used an optical profilometer to measure the thickness of the individual cuticles.

We followed the protocol described in ref. 19 to conduct the uniaxial tensile tester for UltraThin films (TUTTUT) to measure the force–displacement responses of the cuticle. The TUTTUT cantilevers were calibrated before each set of measurements, following protocols previously published.^19^ We floated the cuticle films on a water bath. A silicon wafer was dropped on the grip section of the film. The water level was lowered, and the wafer positioned into the clamp and rigidly fixed onto the walls of the bath container. The other side of the cuticle film was attached to a cantilever (an aluminium-coated cover glass). The grip section wafer was set to be parallel to the bottom edge of the cantilever. The stage connected to the water bath container was then pulled at a constant velocity of 0.2 mm s^−1^. A laser reflective system recorded the cantilever deformation, which was then used to calculate the resulting force on the cuticle film. For each petal developmental stage, we analysed three isolated cuticle films. We used the force–displacement responses to determine the average stress–strain responses of the cuticle samples. The strain was calculated by dividing the applied far-field displacement by the initial sample length. Since the shapes of the isolated cuticle samples were irregular (*i.e.*, not rectangular nor standard geometries, such as dog bone shapes), we used finite element analysis (FEA) with the same geometries as the experiments to calibrate a geometric factor for each experiment to determine the Young's modulus of the cuticles (Fig. S5). Using FEA to calibrate the stress–strain response has been used previously for inhomogeneous deformation fields.^20^ Specifically, the Young's modulus of the FEA model was adjusted until the relationship between resultant force and applied displacement matched experimentally measured trends for each sample geometry. We then obtained the nominal cross-sectional area in the loading direction *A* by *A* = *kL*/*E*, where *k* is the experimentally measured stiffness, *L* is the gauge distance between load grippers, and *E* is the determined Young's modulus. The stress was then determined by dividing the resultant force by the nominal cross-sectional area, *F*/*A.*

### Histology

To assess the effect of removal of cuticle proper on integrity of the cuticular layer below, the cuticle proper was removed from the petals of stage 5 flowers using a sharp, flexible razor blade under the microscope. Petals with intact cuticle proper were included as controls. Samples were infiltrated with 1X PBS and embedded in 4% agarose. Thin cross-sections were generated by hand under the microscope. The sections were stained with Toluidine blue (0.05%) for 1 minute, then washed with 1X PBS for 1 minute. Bright field pictures were taken with a VHX-5000 Keyence microscope.

## Results

### Characterisation of growth of *Hibiscus trionum* petal layers

**Fig. 1:**
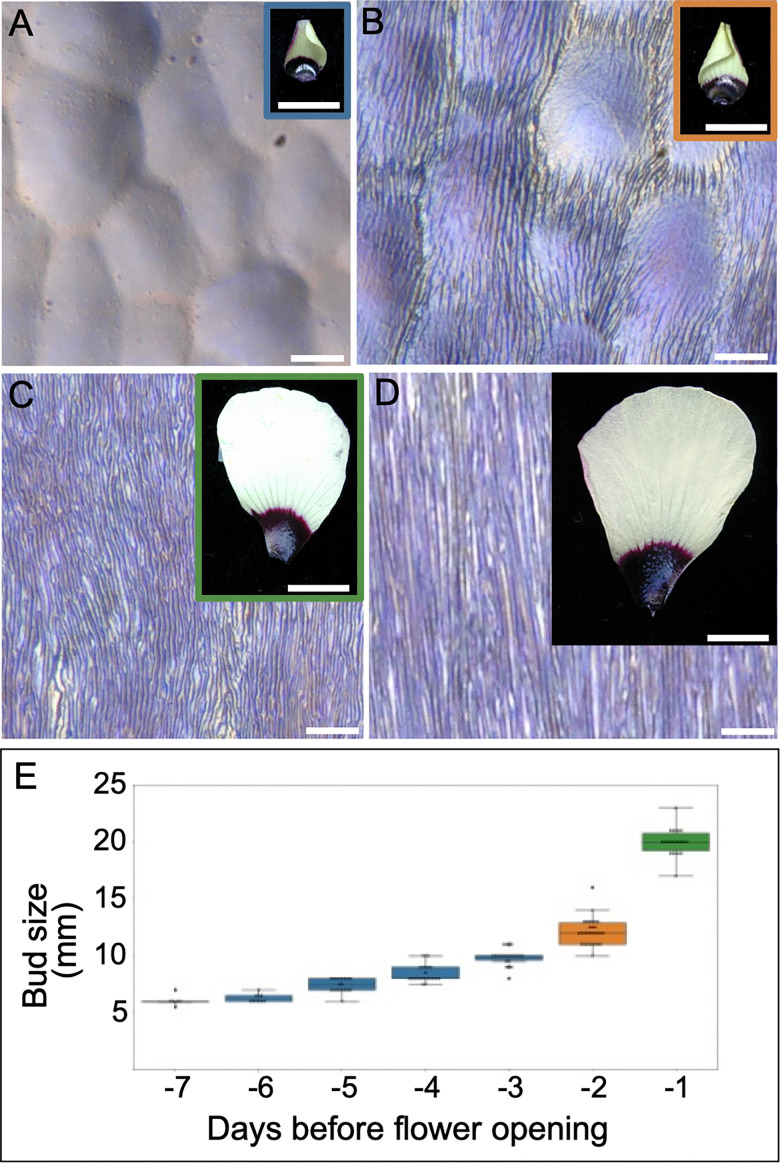
Petal growth and striation formation in *Hibiscus trionum*. (A) A 10 mm flower bud petal with smooth petal epidermis (pre-striation stage). (B) 12 mm flower bud petal with striations starting to form. (C) Mature flower bud petal with striations formed across the purple area of the petal. (D) Open flower petal with fully developed semi-ordered striations. (E) Flower bud size (without epicalyx) from 7 days before opening. Pre-striation stages (−7 to −3 days) are indicated in blue bars, the striation onset stage (−2 days) in orange, and the mature bud stage in green; these colours indicate stage in the petal images in A–D. Scale bars represent 10 μm in A–D, and 10 mm in the insets.

Petal development in *H. trionum* involves changes in size, shape, colour, and texture. The cuticular striation patterns in the purple-coloured section of *H. trionum* petals take around 3 days to develop, immediately before flower opening. During this time the flower bud also increases its size dramatically.^21^Fig. 1A–D summarizes cuticular striation development as a function of time. Over three days, a bud (initially less than 10 mm in length) develops from a pre-striation stage displaying smooth petal cuticle, through a striation onset stage, to a mature bud (around 13 mm in length) which displays ordered striations, and finally to a fully developed and open flower. The striations are only formed on the purple-coloured part of the petal - the white region of the petal is composed of epidermal cells with a conical shape and a smooth cuticle that does not form striations.^21^

**Fig. 2:**
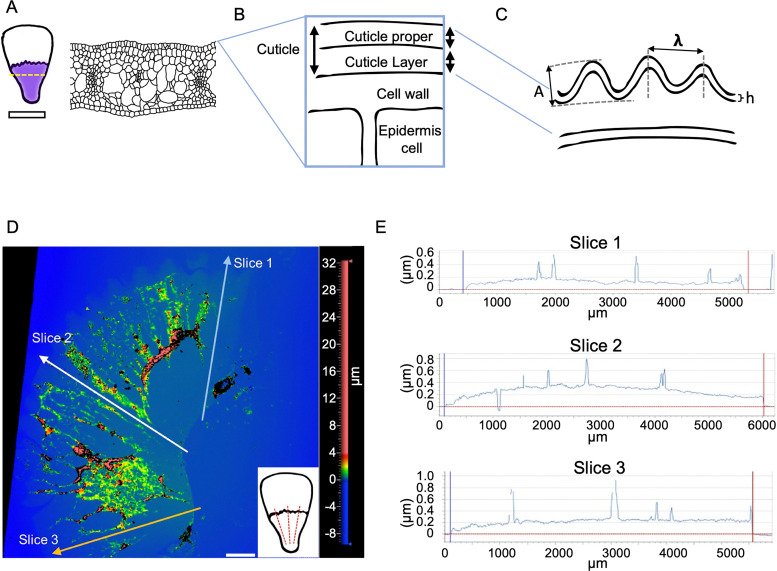
Cuticle thickness analyses. (A) Cuticle striations are formed on the epidermal cells in the purple region in the petals. (B) Schematic representation of cuticle and its parts: cuticle proper (cutin + wax) and cuticle layer (cutin + wax + pectins). (C) Features of the wrinkles, A, amplitude; *λ*, wavelength; h, film thickness. (D) Profilometer analysis of cuticle proper removed from the petal. (E) Thickness was measured in 3 slices across the petal represented by the red dashed lines in the bottom right inset in (D). Scale bar represents 1 mm in D.

To design protocols for accurate indenting of cuticle layers using AFM, we required a detailed understanding of cuticle thickness. We have previously measured the thickness of the cuticle proper (the upper layer) at different stages of petal development from cryoSEM fracture images of the entire layered structure, taking measurements from both the crest and the base of the ridges^4^ (also summarized in Table 1). Here, to measure the variation in thickness of the cuticle proper across the entire petal surface, we used an optical profilometer (Zygo, Nextview NX) with our isolated cuticle preparations (Fig. 2). The thickness of the isolated cuticle proper mostly varied from 150 nm to 350 nm across the petal at the open flower stage (Fig. 2E), similar to the range of measurements recorded from non-isolated layered images (Table 1). However, in some regions we recorded peaks as thick as 800 nm, with greatest thickness in the central part of the purple region (Fig. 2E). We therefore focus on the central part of the purple region of the petal, where striations first develop.

We used data from cryoSEM fracture images^4^ to perform a detailed characterisation throughout development of the thickness of the substrate, the cuticular layer, again to inform mechanical measurements. The cuticular layer increases in thickness during striation onset and through early striation development (Table 1), then it shows a slight reduction in thickness as the petal grows rapidly at the final stage of bud development (Fig. 1E and Fig. S6). This observation is consistent with rapid cell surface growth, accompanied by significant mass increase, during the final stage of petal development.

In summary, growth of the *H. trionum* petal involves changes in the thickness of both layers of the cuticular bilayer that forms the striations, and that thickness was quantified to inform mechanical analyses.

### Characterisation of Young's moduli of the cuticle proper and the cuticular layer

A number of papers have suggested that the cuticular striations of *H. trionum* develop as a result of mechanical instability,^22,23^ with our latest model^4^ proposing that their formation is the result of differences in the mechanical properties of two layers within the cuticle: the cuticle proper (top layer) and the cuticular layer (bottom layer). The model tested a number of values of stiffness ratio between the two layers and defined a range suitable for the formation of striation patterns. However, to test this model it was necessary to develop a method to define the Young's modulus of the layers of living tissue.

**Fig. 3:**
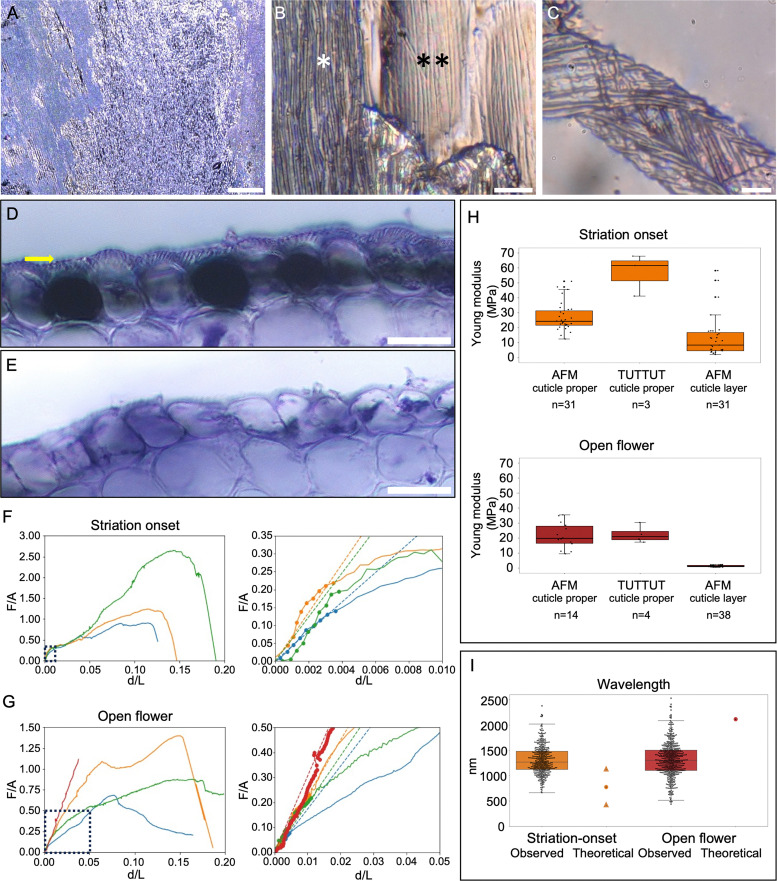
Characterisation of stiffness of cuticular layers during striation in *Hibiscus trionum* petals. (A) and (B) Keyence images of a petal before and after cuticle proper removal. (B) Overview of a petal region after cuticle proper removal, cells still with the cuticle proper are indicated with *, cells without the cuticle proper are marked with **. (C) Isolated cuticle proper after being removed from the petal. (D) Cross section of an intact wild type petal (purple region, open flower), and (E) a petal after cuticle proper removal. Yellow arrow indicates the cuticle proper. (F) and (G) Stress vs strain values from the uniaxial tensile tester for ultrathin films (TUTTUT) measurements of the cuticle proper at the striation onset (F) and at the open flower stage (G). The left subplots show the full strain range responses and the right subplots are the zoom-in at the small strain regime indicated with dotted line boxes. The three samples of the onset stage have the moduli of 41.2, 67.9, and 61.6 MPa respectively. The four mature stage samples have the moduli of 17.3, 22.5, 19.3, and 30.4 MPa respectively. The fitting regime for linear elasticity is marked as dots, and the dashed lines are the linear fittings. For striation onset specimens, the fitting was based on the first 0.4% of average *d*/*L* values. For open flower specimens, the fitting was based on the first 1% of average *d*/*L* values. (H) Young's moduli values calculated from atomic force microscopy (AFM) and the TUTTUT measurements during striation onset and at the open flower stage. The box delimits the interquartile range (IQ3 minus IQ1), the horizontal line depicts the median, and the whiskers indicate the maximum (Q3 + 1.5(IQR)) and the minimum (Q1 − 1.5(IQR)); there are some outliers. (I) Observed vs theoretical wavelength of striations. Wavelength theoretical values were calculated using our measurements and eqn 1, values for observed wavelength in Hibiscus petals were reported in ref. 4, the circle indicates the predicted value using the average values for film thickness and Young's modulus of the film and substrate, the triangles indicate the predicted values considering the standard deviation for film thickness, and Young's modulus values. Scale bars: (A) 100 μm, (B) 10 μm, (C) 5 μm, (D) and (E) 20 μm.

In this work, as in other analyses of living materials,^14,24^ we treated the cuticle as an isotropic material, thereby inferring information on the in-plane properties of the materials from measurements on the z-component. We used atomic force microscopy (AFM) and the uniaxial tensile tester for UltraThin films (TUTTUT) to measure Young's moduli of the cuticle proper, comparing data from the two very different approaches with different operators in different labs to develop confidence in the approach taken for AFM analysis of this layer. We used AFM to take similar measurements from the cuticular layer following the removal of the cuticle proper (Fig. 3). Fig. 3B shows the appearance of the petal after removal of the cuticle proper, revealing the cuticular layer. Cross sections of the samples are shown in Fig. 3D and E. Fig. 3C shows the appearance of an isolated cuticle proper, used for TUTTUT experiments.

The results of our AFM experiments to measure the cuticle proper (upper layer) stiffness are summarized in Fig. 3H and Fig. S7 for striation onset and open flower stages. These measurements were taken by shallow indentation within the depth range of the cuticle proper, without any dissection or perturbation of the layer structure. The mean Young's modulus of the cuticle proper at striation onset measured using this approach is around 27.06 MPa, decreasing to around 21.57 MPa at the open flower stage (Table 1).

In parallel, we measured the Young's moduli of isolated cuticle thin films through TUTTUT.^19,20^ The results of these experiments to analyse the cuticle proper (upper layer) are summarized in Fig. 3F and G for striation onset and open flower stages. The mean Young's modulus of the cuticle proper at striation onset measured using this alternative approach is 56.90 MPa, decreasing to approximately 22.40 MPa at the open flower stage (Table 1).

Young's modulus of the cuticular layer (substrate layer) was measured using AFM on intact petals from which the upper cuticle layer had been removed mechanically. This approach left upper cell walls, including the cuticular layer, and all other tissue layers, intact (Fig. 3D and E). The mean Young's modulus of the cuticular layer at striation onset is 13.65 MPa, decreasing to around 1.35 MPa at the open flower stage (Table 1). This significant decrease in stiffness of the cuticular layer during tissue development has not previously been described.

All of our measurements indicate that the cuticular layer (substrate) is softer than the cuticle proper (upper layer), as hypothesized in previous studies.^3,4,25^ The difference in the stiffness between these two layers increases through developmental time, as the cuticular layer becomes softer during petal growth to a much greater degree (approximately 10-fold) than does the cuticle proper (approximately 1.3 to 2.5-fold, depending on method of measurement).

The initial linear slope of the stress–strain response indicates Young's moduli of the cuticles (Fig. 3F and G). While the linear relationship upon loading may indicate that the substrate stiffness is significantly greater than the cantilever stiffness, we note that cantilever bending has been accounted for in the analysis and that the measured mechanical properties for the specimens is consistent with an independent measurement method, referred to as TUTTUT. We found similar moduli values with both techniques in the open flower, whereas at the onset stage we observe a difference in the values obtained from the two techniques (Fig. 3H), which we consider in the Discussion.

### Wavelength and critical strain in *Hibiscus trionum* petal striation patterns

The striation patterns observed on the *H. trionum* petal cuticle have key features, such as the amplitude (A) and wavelength (λ) (Fig. 2C). To characterize the pattern wavelength (the distance between two adjacent crests), we used previously published detailed characterisation of *H. trionum* cuticle material properties (*i.e.*, cuticle film thickness, *h*),^4^ at different stages in combination with the Young's moduli presented here (Table 1). We verified the relationship between stiffness and thickness of our biological materials and wrinkle wavelengths, using eqn (1) to predict the theoretical wavelength. With the measured Young's moduli using AFM data and the measured thickness at the striation onset stage, we calculated a theoretical wrinkling wavelength of 799 nm (Fig. 3I and Table 1), which increases at the mature bud stage (1602 nm), then to 2117 nm at the open flower stage. The predicted values for the wavelength at the striation onset stage vary from 1246 to 430 nm (Fig. 3I) when variation in the cuticle proper thickness and stiffness of the layers are considered (standard deviation: 25.7 nm for cuticle proper; 9.40 and 13.84 MPa for the cuticle proper and cuticle layer respectively). In comparison, the mean measured values for the observed wavelength are 1308 nm at the onset stage (ranging from 600 nm to 2400 nm), 1289 nm at the mature bud (ranging from 500 nm to 2200 nm), and 1313 nm (ranging from 400 nm to 2500 nm) in the open flower stage.^4^

The predicted wavelengths we obtained were similar to those that we measured (Table 1), and within the range of variation seen in nature. However, at the open flower stage the differences between theoretical and observed wavelengths were more significant than at the earlier flower stages.

## Discussion

Here, we analysed stiffness of a layered living tissue during development to understand the formation of striation patterns on the epidermal surface in *H. trionum* petals. We describe AFM and TUTTUT techniques, which generate reasonably consistent data, providing confidence in the methods applied. We propose that our AFM protocol, using morphology-informed indentation depths and mechanical removal of upper layers, is sufficient to allow reasonable estimates of the Young's moduli of the layers of layered living tissues.

The use of AFM to calculate Young's modulus is challenging in a number of ways. First, it assumes that the material we measure is both isotropic and incompressible. This may well not be an accurate representation of the complexity of biological materials, but it is a necessary constraint of current approaches to exploring their mechanical properties.^6,13,26^ When applying the technique to a layered system, the depth of indentation and the choice of indentation shape and cantilever size present additional challenges and can result in errors in the calculations. However, our use of TUTTUT to acquire estimates of Young's moduli by an alternative approach, only suitable for the upper layer and not readily available to biological labs, provided a means of assessing the validity of our AFM data. The fact that our AFM Young's moduli are similar to the values obtained with TUTTUT (Fig. 3H) suggests that we can accurately measure petal cuticle stiffness with AFM and provides confidence in our cuticular layer data by extension. We note that the TUTTUT and AFM data for cuticle proper stiffness at the open flower stage are remarkably similar (means of 22.40 MPa and 21.57 MPa, respectively). We consider this convergence of values remarkable given the extreme differences in tissue treatment prior to the measurement, the different techniques applied, and the additional variable that measurements were made on different individual plants grown in different glasshouses on different continents. The data for cuticle proper stiffness at striation onset are more distinct (means of 56.90 MPa from TUTTUT and 27.06 MPa from AFM, Table 1). However, even these values are reasonably consistent, given the variables already mentioned and the fact that experimenters in different labs had to independently select petals at this key developmental stage (inherently more error-prone than the objectively simple fully open flower). We also note that striation onset does not occur synchronously across the entire purple region of the petal but begins at foci often associated with the edges of cells or other perturbations in the tissue surface. It is likely that the mechanical properties of the petal vary across the surface at this striation onset stage. Our AFM approach necessarily uses small pieces of tissue, potentially adding further variability to the measurements we recorded. Nonetheless, the finding that both approaches generated Young's moduli values within the same order of magnitude provides further confidence in the AFM approach.

Our data indicate the importance of taking AFM measurements at appropriate stages of development, as living tissues change their mechanical properties with time. We measured the stiffness in the cuticular layers over time to calculate the stiffness ratio (cuticle proper/cuticle layer) between them and to explore whether it is sufficient to generate the observed striation patterns in *Hibiscus trionum*. According to our data, the stiffness of the cuticle layers changes over developmental time, with both layers becoming softer at the open flower stage than they were at the striation onset stage. However, of the two layers it is the (lower) cuticular layer for which stiffness decreases most significantly. As a result, the stiffness ratio of the materials increases dramatically: at the onset of striation initiation, it is around 2 and at the open flower stage it has increased to approximately 16 (based on AFM data). Future analyses will focus on the consequences of changing the timing of cuticle formation and cuticle stiffness changes during petal development.

The stiffness of cell layers can influence plant development and affect the shape and structure of an organ. Some changes in tissue mechanical properties (including stiffness) have previously been shown to occur during plant development, affecting cell division and expansion. In Arabidopsis roots, differential cell wall stiffness is associated with the elongation or differentiation zones.^27^ In Arabidopsis fruits, the stiffness of the outer layer in the valves is constant during fruit elongation, however, a mutant affected in the degradation of cell wall components displayed an increase in fruit stiffness, affecting fruit elongation.^28^ In the Hibiscus petals, we observed a decrease in stiffness of both the cuticle proper and the cuticular layer over time. With respect to the cuticle proper, this phenomenon could be related to exposure of the petal surface to the environment: the flower bud is closed during development, then opens and is exposed to the air with differences in humidity and temperature that might influence cuticle chemistry and mechanical properties. For the cuticular layer, we hypothesise that the even greater degree of softening that we recorded might reflect mechanical changes to the cell wall as rapid cell expansion occurs during petal growth. Cell expansion is dependent on expansin-induced loosening of bonds between cellulose and hemicellulose molecules,^29^ which might be expected to reduce the stiffness of a material. Alternatively, given that the Hibiscus flower is open for a relatively short time (approximately 2 hours), it is possible that the softening we record in both layers represents the onset of senescence, involving changes in cell metabolism and turgor.

Previous studies of plant surface stiffness have largely treated the cuticle and cell wall as a single material, rather than the layers examined here. However, previous studies have explored the elastic moduli of isolated cuticles from tomato fruits, including during the growth of the tomato.^30,31^ The tomato fruit cuticle is extremely thick (ranging from 5.8 to 15.7 μm)^32^ and provides protection from water loss and pathogen infection, thereby allowing fruits to be transported and sold for significant time windows post-harvest. The cuticle proper in our Hibiscus petals is soft (23 MPa and 21.57 MPa for TUTTUT and AFM respectively, open flower) compared to published values for tomato fruit (ranging from 60 to 360 MPa).^32^ This big difference in Young's modulus between cuticles in petals and fruits could be related to the different functions of cuticle in different organs.^17,32^ In fruit the cuticle acts as a barrier to protect against dehydration, pathogens, and insect pests over a much longer time than the lifespan of a petal.^33^

Although we observed changes occurring at the whole petal level during development, such as increases in cell size and layer thickness, we note that the striation patterns appear to be stable once they have formed. Lugo *et al.*^4^ also previously showed that the striation wavelength is set when the pattern emerges and remains constant throughout petal growth. This suggests that upon striation formation, the deformation of the tissue seems to be locked despite the changes in cell size we observe from striation onset to flower opening. We hypothesise that this stability of the wrinkling wavelength across petal development could be an effect of the inelastic nature of biological materials during growth. This hypothesis is consistent with our observations after the isolation of the cuticle proper (mechanically or with enzymatic treatment), where the wrinkling patterns were still preserved in both the isolated cuticle proper and in the cuticular layer below (Fig. 3A–C). The onset of inelastic mechanical responses at larger strains is also consistent with the measured TUTTUT data (Fig. 3F and G) that show nonlinear responses at larger displacements.

When our data collected using AFM are applied to the theoretical model of Lugo *et al.*^4^ we find that the predicted value for the wavelength of the striations is consistent with the range of actual wavelength values measured on the petals, but not consistent with their mean or median. The living petals have striations with a mean wavelength of approximately 1300 nm, while our predictions using the data obtained here would be for a wavelength of around 800 nm at striation onset, increasing to around 2000 nm as the flower matures. Our hypothesis is that this discrepancy is generated by changing properties of this living system during development. During the striation onset stage, large parts of the flower are not yet producing striations. Given the very small surface measured with AFM, we could well be measuring the properties of parts of the tissue that are not yet capable of undergoing wrinkling. This is also suggested by a bigger spread of the AFM data and a bigger discrepancy between the AFM and TUTTUT measurements in the cuticle proper at the striation onset stage compared with the cuticle proper of the open flower. Additionally, the wavelength prediction using eqn (1) only applies to a flat domain *i.e.*, the tissue at the striation onset stage. At the later stages, when the cell surface and the petal surface are more curved, we observed bigger differences between the theoretical *vs.* the observed values. The simulations performed by Lugo *et al.*^4^ have shown how curvature can delay wrinkling onset and therefore could result in a longer wavelength than the one predicted by the simple equation. The stiffness values of the cuticular layer at the mature bud stage measured with AFM are greater than the values for the open flower (Table 1). At the same time the cuticle thickness has increased relative to the onset stage, and the cuticle stiffness has decreased only a little relative to the onset-stage, therefore the theoretical prediction of the wavelength increases. This suggests that during the night that divides the final two growth stages and sees the flower open fully in the morning (Fig. 1C–E both the growth of the layers and the softening of the substrate may result in the right material properties to develop the wavelength observed in the flowers. The dynamic nature of this system and the presence of a curved surface may explain the range of wavelengths observed in the flower. It is ultimately this imperfection in the pattern that creates the blue halo that increases salience to pollinators.^2^

## Conclusions and perspectives

Here, we report the measurement of stiffness of cuticular layers through petal development. According to our analyses, the AFM and TUTTUT measurements overlap, indicating that both methods could be used to measure stiffness of cuticular layers in *Hibiscus* petals and in other living bilayer systems. Since AFM is more accessible for biological labs, we propose that our AFM protocol, taking into account depth of indentation, is sufficient to provide reliable data on stiffness of layered living systems. TUTTUT, a newly developing method, could provide future insight into larger strain mechanical responses, including the onset of inelastic behaviour.

The characterisation of *Hibiscus trionum* petals using multiple approaches has allowed us to describe the cuticle bilayer in the flower and the buckling phenomenon under the effect of compressive forces. We show that the equation used to describe materials reliably describes our biological system and we can predict the wavelength after buckling to a reasonable degree of accuracy by knowing the thickness of the film and the stiffness of the two layers. *Hibiscus trionum* petals, despite being a living and changing biological system, behave and buckle like other materials.

Our study also reveals the dynamic development of material properties, with tissue stiffness changing through developmental time. This dynamic property may allow multi-functionality that is difficult to achieve through a static material and may be of interest in the bioinspired design of new materials.

We aim to use the described protocol to dissect the contribution of genetic, biochemical, and environmental factors in the formation of softer or stiffer cuticular layers and their influence in the formation of cuticular patterns. Additionally, further analysis will be conducted to assess the contribution of other cellular features, such as cell curvature and cell shape, to the formation of the observed wrinkling patterns.

## Author contributions

Conceptualisation: CAA, CC, AJC, BJG; methodology: CAA, CC, HF, AJC, BJG; investigation: CAA, CC, HF; validation: CAA, CC, HF, CAL, UP; formal analysis: CAA, CC, HF, CAL; resources: AJC, BJG; writing – original draft: CAA, HHU; writing – review & editing: HHU, AJC, BJG; visualisation: CAA, CC, HHU, HF, UP, CAL; supervision: CAA, CC; project administration: AJC, BJG; funding acquisition: AJC, BJG.

## Conflicts of interest

The authors declare no competing interests.

## Supplementary Material

SM-021-D4SM01406E-s001

## Data Availability

The raw data that support the findings of this study are available from the corresponding author, Beverley J. Glover, upon request. The data supporting this article have been included as part of the supplementary information (SI). Supplementary information: Overview of the tip used in the atomic force microscopy experiments; atomic force microscopy maps; examples of force-displacement curves obtained in the AFM experiments; examples of data processing in the AFM experiments; finite element calibration of TUTTUT experiment; *Hibiscus trionum* petal mass increase over time; comparison of Young's modulus values in the cuticle proper. See DOI: https://doi.org/10.1039/d4sm01406e.
