# Supplementary material for: Characterisation of cuticle mechanical properties: analysing stiffness in layered living systems to understand surface buckling patterns
Source: Soft Matter. 2025 Oct 8;21(42):8231–41. doi: 10.1039/d4sm01406e (PMC12522587; doi:10.1039/d4sm01406e)
Supplement: SM-021-D4SM01406E-s001 [file SM-021-D4SM01406E-s001.pdf]

This journal is © The Royal Society of Chemistry 2025

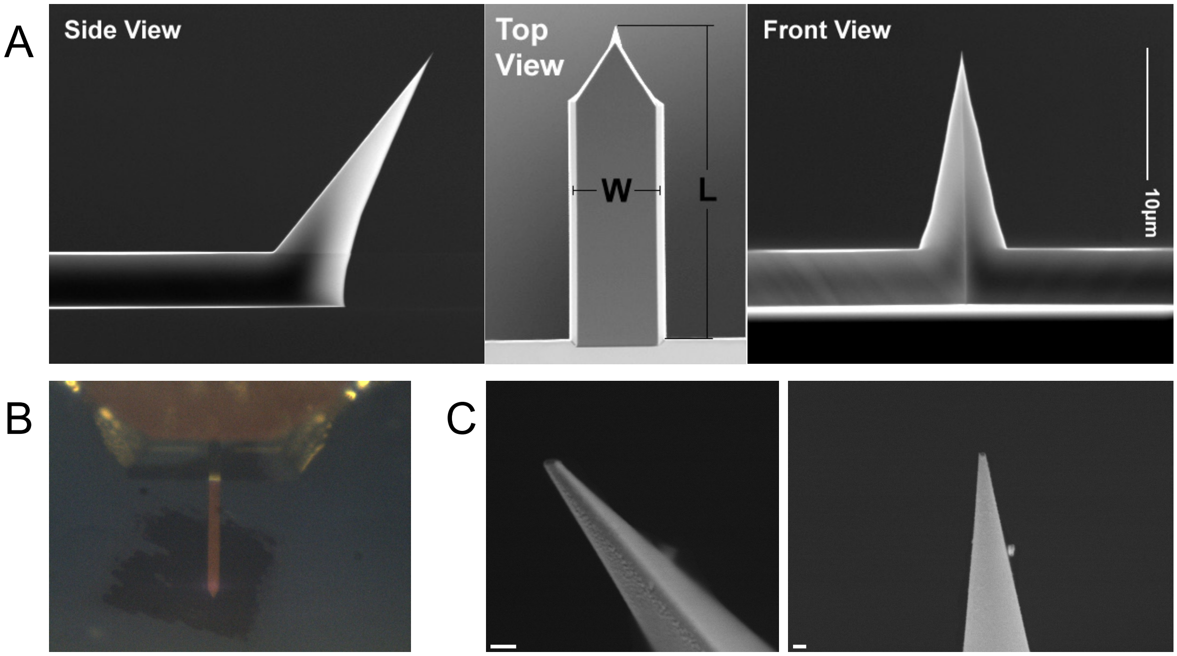

Supplemental Figure 01. Overview of the tip used in the atomic force microscopy (AFM) experiments. A) Side, top, and front view of the tip. Images provided by the manufacturer. B) Top view of the tip taken with the AFM built-in camera or C) with the scanning electron microscope. Scale bars in C represent 200 nm.

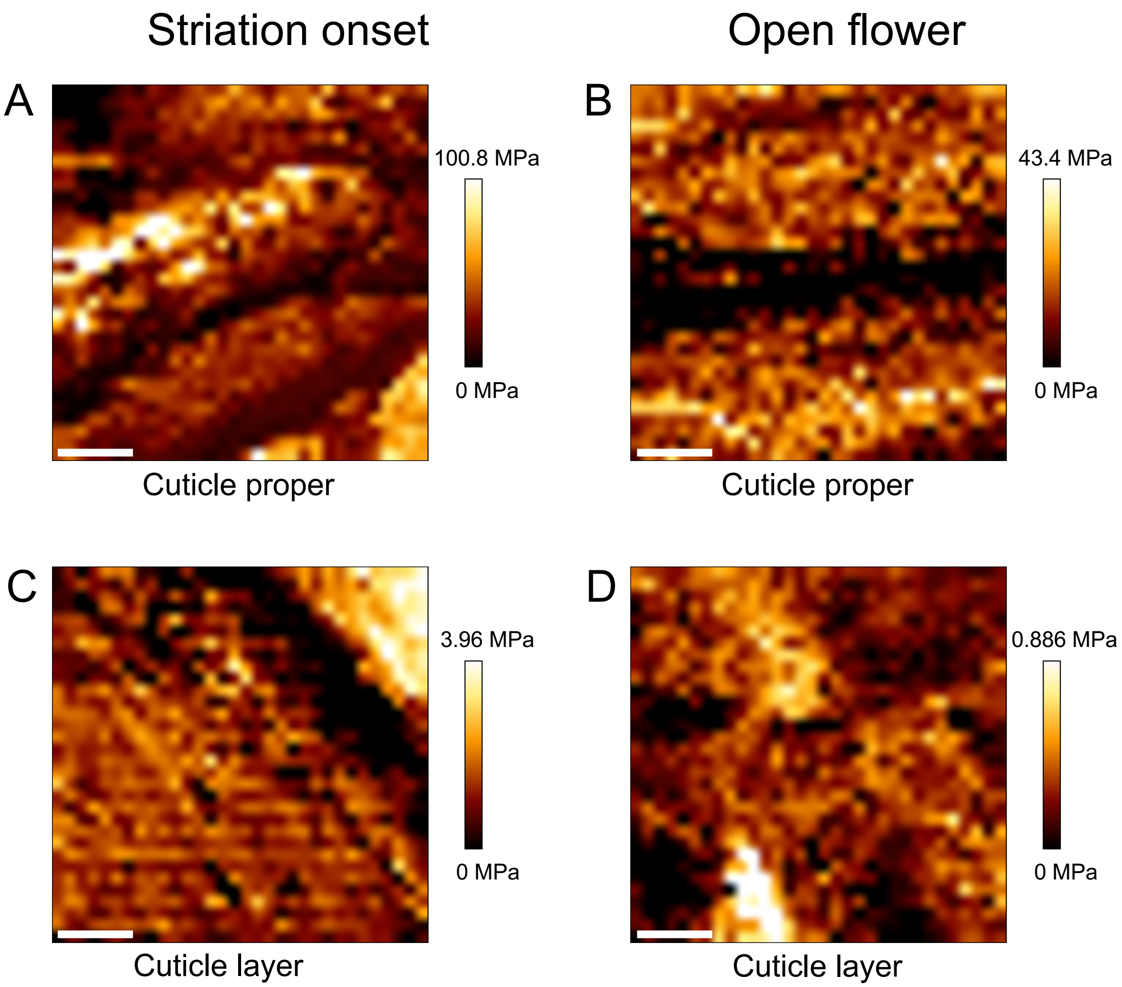

Supplemental Figure 02. Atomic force microscopy (AFM) maps showing Young’s modulus for the cuticle proper (A, B) and cuticle layer (C, D) at the striation onset and at the open flower stage. Scale bars represent 200 nm.

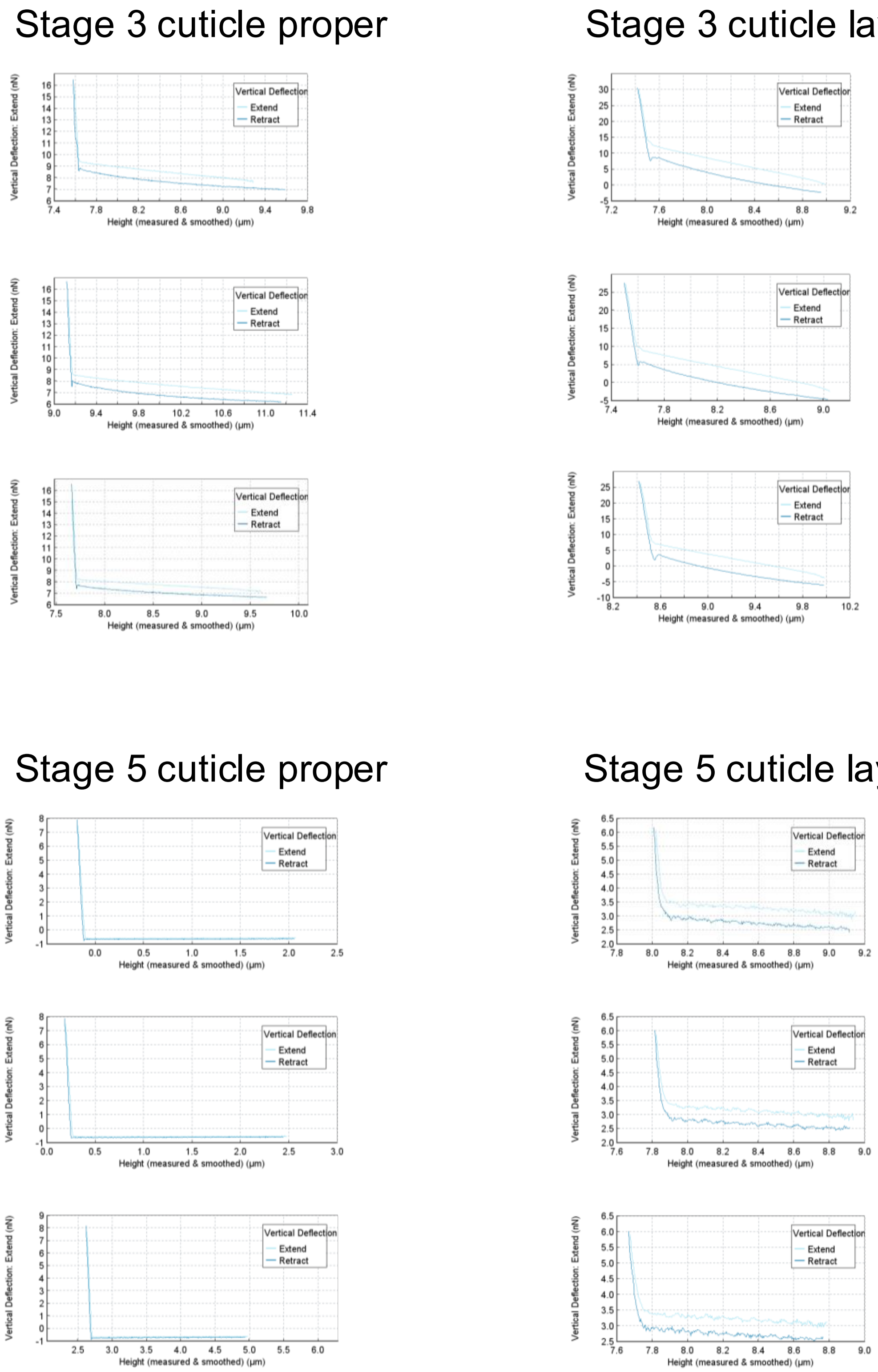

Supplemental Figure 03. Some examples of force-displacement curves obtained in the AFM experiments (raw data). These 12 representative examples were selected from 100s recorded during the data capture.

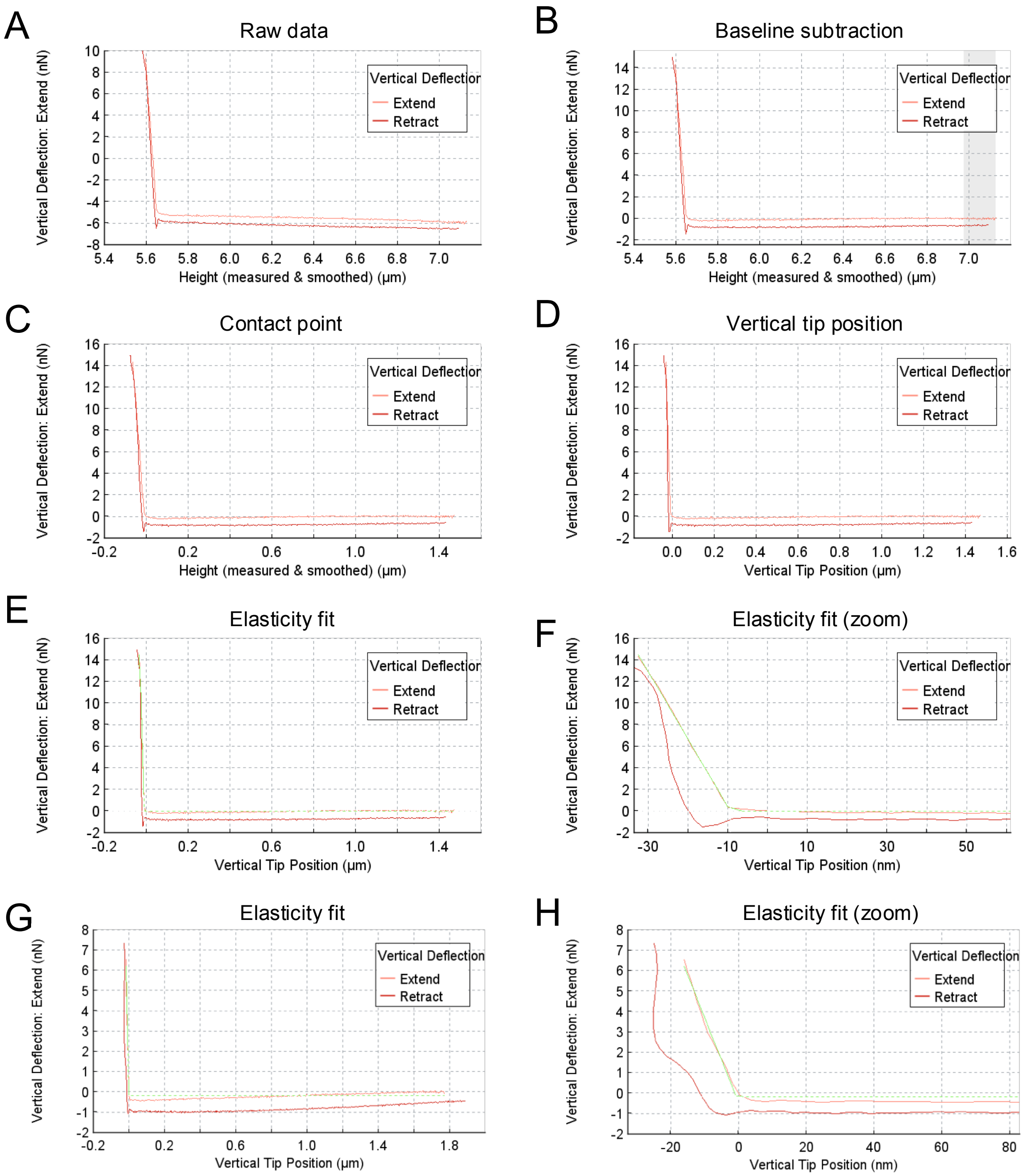

Supplemental Figure 04. Some examples of data processing in the AFM experiments. A) Raw data (Force-displacement, approach curves, light red) were processed with the JPK data processing software (JPK Bruker v 6.4.-1+), data was processed with the following operations: B) baseline subtraction, C) determination of contact point, and D) adjustment of vertical tip position. Young's modulus was obtained by fitting the curves (green lines on the extend curves) with a contact mechanics model described in the main text, E-F) shows the curves for cuticle layer at stage 3 petals, and G-H) for cuticle proper. These graphs are a representative example from 100s recorded during the data capture and processing.

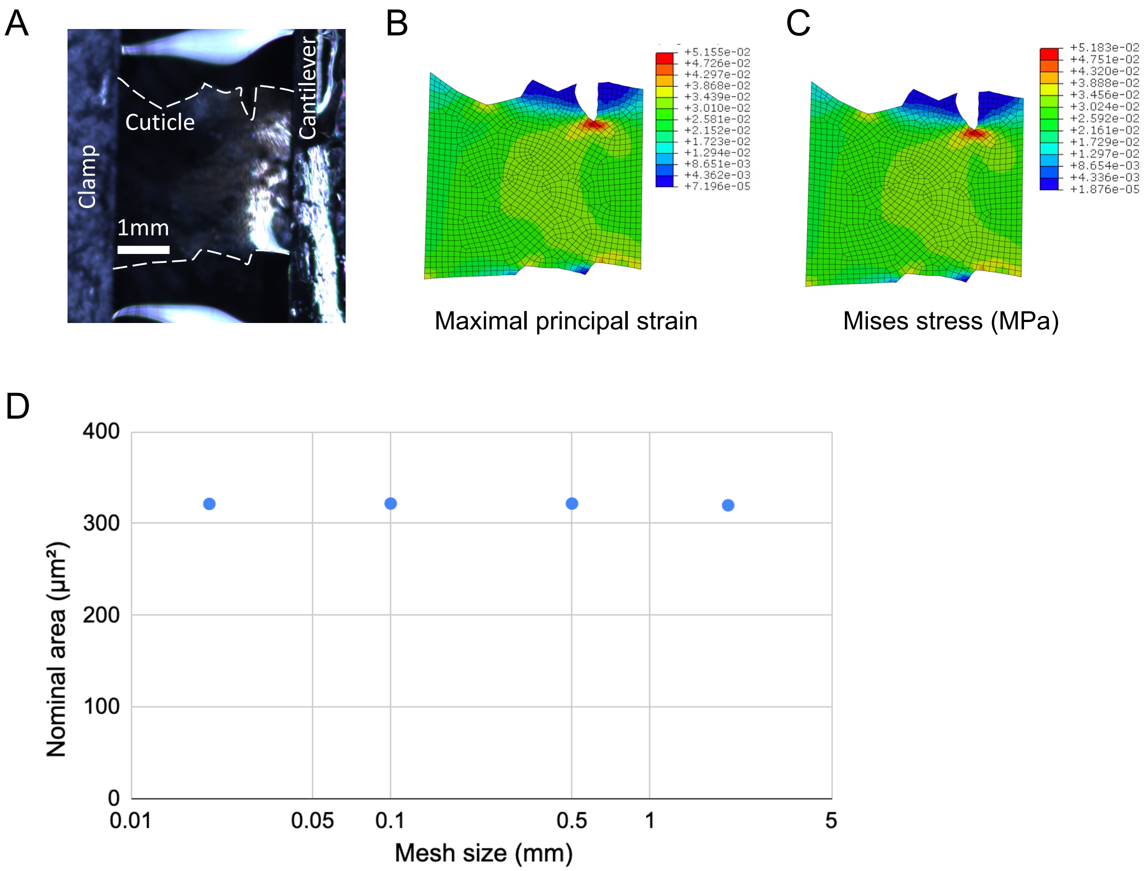

Supplemental Figure 05. Finite element calibration of TUTTUT experiment. A) TUTTUT experiment on a petal cuticle in the striation onset stage. The floated cuticle layer was adhered to a cantilever at right side and a loading clamp at left side, subject to a tension in horizontal direction. Linear elastic finite element calculation on a cuticle model with the same shape and thickness. B) The maximal principal strain distribution and C) the von Mises stress distribution with an overall strain of 2 %. D) The mesh sensitivity study with mesh from 0.02 mm to 2 mm. The calculated nominal area has 0.57 % variation.

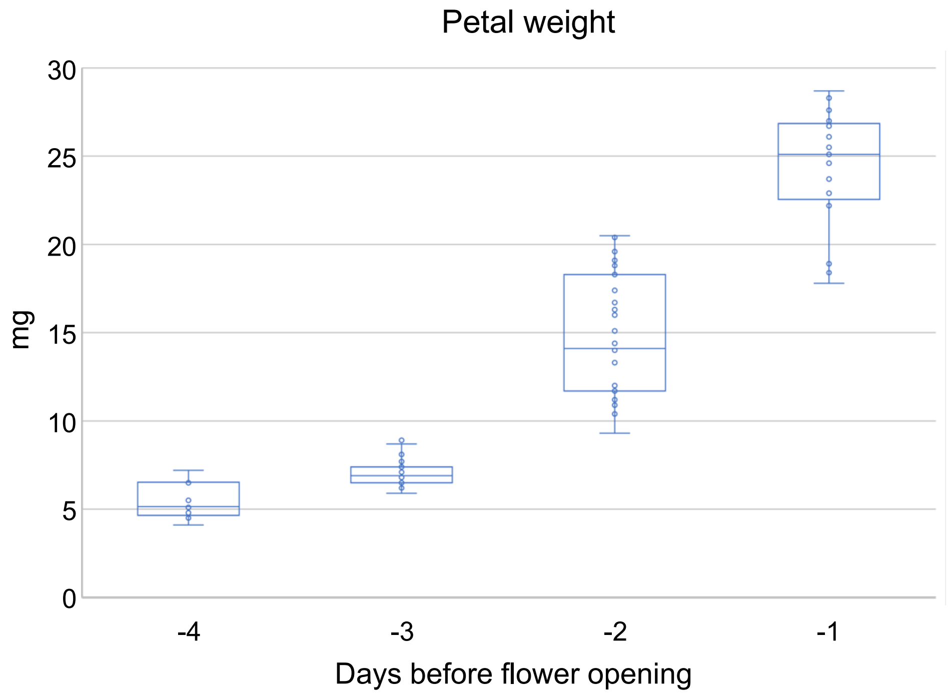

Supplemental Figure 06. *Hibiscus trionum* petal mass increase over time. Weight of single petals from flowers before opening. Bud size was used to estimate time before opening.

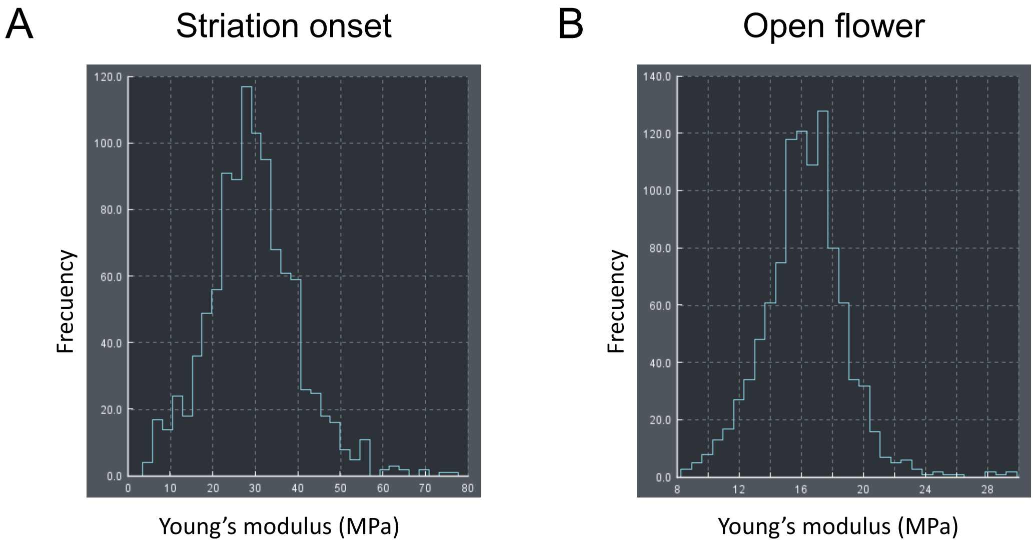

Supplemental Figure 07. Comparison of Young's modulus values in the cuticle proper in *H. trionum* petals. One example of the Young's modulus values measured with Atomic Force Microscopy at the striation onset and at the open flower stage.
